# Spectroscopic Behaviour of Two Novel Azobenzene Fluorescent Dyes and Their Polymeric Blends

**DOI:** 10.3390/molecules25061368

**Published:** 2020-03-17

**Authors:** Rosita Diana, Ugo Caruso, Stefano Piotto, Simona Concilio, Rafi Shikler, Barbara Panunzi

**Affiliations:** 1 Department of Agriculture, University of Napoli Federico II, NA 80055 Portici, Italy; rosita.diana@unina.it (R.D.); barbara.panunzi@unina.it (B.P.); 2 Department of Chemical Sciences, University of Napoli Federico II, 80126 Napoli, Italy; 3 Department of Pharmacy, University of Salerno, SA 84084 Fisciano, Italy; piotto@unisa.it; 4 Department of Industrial Engineering, University of Salerno, SA 84084 Fisciano, Italy; sconcilio@unisa.it; 5 Department of Electrical and Computer Engineering, Ben-Gurion University of the Negev, POB 653 Beer-Sheva 84105, Israel; rshikler@ee.bgu.ac.il

**Keywords:** azobenzene, dye, fluorophore, colorant, polymeric blend

## Abstract

Two novel symmetrical bis-azobenzene red dyes ending with electron-withdrawing or donor groups were synthesized. Both chromophores display good solubility, excellent chemical, and thermal stability. The two dyes are fluorescent in solution and in the solid-state. The spectroscopic properties of the neat crystalline solids were compared with those of doped blends of different amorphous matrixes. Blends of non-conductive and of emissive and conductive host polymers were formed to evaluate the potential of the azo dyes as pigments and as fluorophores. Both in absorbance and emission, the doped thin layers have CIE coordinates in the spectral region from yellow to red. The fluorescence quantum yield measured for the brightest emissive blend reaches 57%, a remarkable performance for a steadily fluorescent azo dye. A DFT approach was employed to examine the frontier orbitals of the two dyes.

## 1. Introduction

Azobenzene derivatives are π-conjugated molecules that have aroused enormous research interest owing to their fascinating characteristics. First, the unique broad UV/Vis absorbance spectra related to the considerable number of vibronic states in each energy level. The color is due to the presence of the N=N chromophore chemical group absorbing light in the visible spectrum. Suitable substituents can achieve the tuning of the color to cover the entire visible spectrum. 

For this reason, from the past until today, azo compounds have been widely used as dyes and pigments. A significant amount of azo dyes is used in manufacturing processes of textile, paper, packaging pharmaceutical, and even food industries [1]. Excellent compatibility with the matrix and chemical and thermal stability are the main requirements. Despite this, photo-catalyzed degradation through illumination by solar light was found in most of the azo pigments, with the resulting discoloration of the dyed substrate.

On the other hand, the ability of azobenzene compounds to reversibly change from the stable *trans*-isomer to *cis* form upon photoirradiation (photoisomerization) causes nonradiative deactivation and very small/negligible fluorescence. The rapid photoisomerization of the azo bridge finds applications in on-off photoswitching techniques, such as in optical data storage, dye-sensitized solar cells, pharmaceuticals, and non-linear optics [2,3,4,5,6,7,8]. Azo-containing molecules can play the role of energy dissipator (quencher) associated with other emitting chromophores, through rapid nonradiative pathways, while the donor is excited for the occurrence of a Förster resonance energy transfer (FRET) [9]. Unlike many chromophores commonly utilized as fluorescent materials, there are a limited number of examples of steadily fluorescent azobenzene compounds.

The structural versatility and the tunable spectroscopic properties of the azobenzene derivatives could be exploited if they are themselves fluorescent. Attempts to increase and to modulate the fluorescence performance of azobenzene compounds are quite recent [10,11,12,13,14,15] and based on preventing fast photoisomerization. This is possible with bulky substituents close to the N=N group [16,17,18,19,20,21] or intramolecular hydrogen bonding preventing the rotation around the nitrogen–carbon bond [19]. The influence of hydrogen-bond interactions on the excited-state dynamics of azo dyes has been examined [22,23]. It appeared that by restricting rotation and isomerization, the excited-state intramolecular proton-transfer (ESIPT) [21,24] lead to emissive azo dyes in solution [10,16,24,25,26,27,28,29]. Conversely, examples of solid-state azo fluorophores are rare in the scientific literature. Those few are typically red emitters [30,31,32,33], particularly advantageous in the context of biological and medical measurements [34,35,36,37]. 

**Scheme 1:**
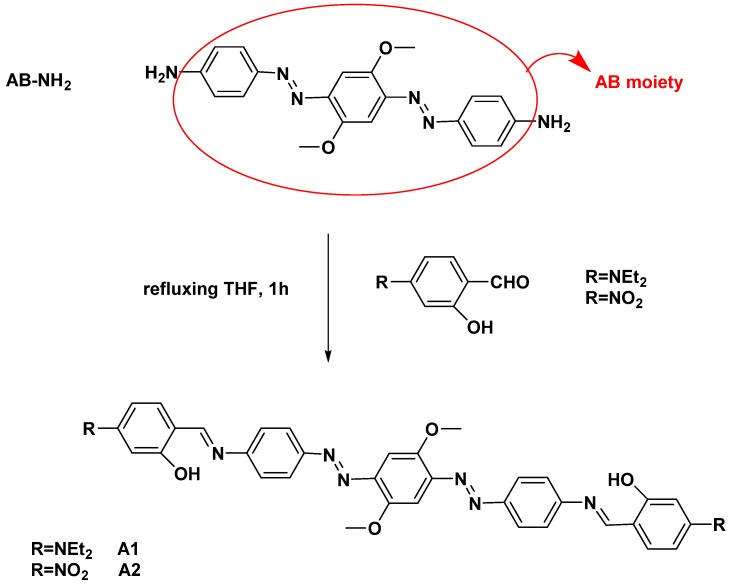
Synthesis and structure of the dyes A1 and A2.

In previous contributions [10,13], we studied azobenzene scaffolds for their unique structural pattern related to their photophysical properties. In this work, two novel azobenzene dyes, A1 and A2 in Scheme 1, were explored. To fulfill the criteria as fluorescent dyes, we have sought stable, processable, and sterically encumbered structures. Based on the same symmetrical bis-azobenzene skeleton (AB in Scheme 1), the two chromophores differ for the terminal substituents, both electron-donor (NEt_2_) or electron-acceptor (NO_2_) groups. DFT computational study was employed to get information on HOMO-LUMO localization at the different conjugated patterns. To impede photoisomerization, we added methoxy substituents on the AB moiety and two terminal Schiff bases ESIPT undergoing sites to restrict molecular rotations.

Under sunlight, the dyes are stable over three months both in solution and in the solid-state, retaining their orange-red color (see CIE: coordinates, International Commission on Illumination, in Table 1 and Table 2). As fluorophores, a significant emission in solution and the crystalline phase was recorded. Different host matrixes were employed to produce polymeric blends from A1 and A2. In addition to classical non-conductive hosts such as polyvinyl chloride (PVC) and poly (styrene) (PS), we also checked conductive, poly (vinylcarbazole) (PVK), and even emissive, poly (9,9-dioctylfluorene) (PFO), polymers. Their coloring ability was examined dissolved in PVC, a white matrix used in many industrial processes. The emission in the solid-state was evaluated in PS, PVK, and PF blends. The last one recently emerged as a useful polymeric matrix for optoelectronic devices [38]. In PFO, the photoluminescence quantum yield (PLQY) of the A1 orange-red blend increases up to an outstanding 57% value.

## 2. Results and Discussion

### 2.1. Synthesis and Optical Behavior of the Dyes and Their PVC Blends

As summarized in Scheme 1, the dyes A1 and A2 were obtained by condensation of the diamino derivative AB-NH_2_ [39] with 4-(diethylamino)-2-hydroxybenzaldehyde and 2-hydroxy-4-nitrobenzaldehyde, respectively. Though structurally similar, a different conjugation pattern is recognizable, D-π-D-π-D respectively for A1 and A-π-D-π-A for A2 (where D = electron donor moiety, A = electron acceptor moiety, and π = conjugated system).

Two ESIPT undergoing sites are generated by the condensation of the amino-terminal groups of the precursor AB-NH_2_ with the salicylic aldehydes, guaranteeing emission in solution. According to a recent approach [26,39,40], it was found that the fast proton transfer in ESIPT sterically encumbered probes are impeded due to restriction of intramolecular rotation (RIR effect), also providing solid-state emission. The central methoxy groups cause steric hindrance on the conjugated skeleton without too much lowering solubility.

Identification and purity degree evaluation were assessed by mass spectrometry and ^1^H NMR. Phase behavior was examined by optical observation and DSC/TGA analysis. All materials are thermally stable up to 330 °C under nitrogen flow with high melting points. The two dyes are soluble in most organic solvents, such as acetone, tetrahydrofuran (THF), methylene chloride, tetrachloroethane (TCE), dimetilformammide (DMF), 1-metil-2-pirrolidone (NMP), and dimethyl sulfoxide (DMSO). Their absorption and emission maxima, in solution and the solid films, are reported in Table 1. 

**Figure 1:**
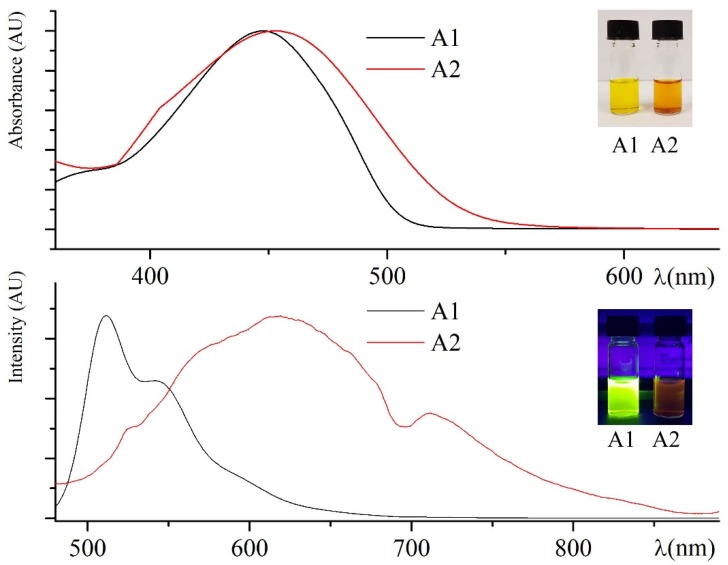
Absorption (above) and emission (below) curves of A1 (black curves) and A2 (red curves) in THF solution. The same samples in natural light (above) and under UV lamp at 365 nm (below) in the insets.

In Figure 1, absorption and emission curves of A1 and A2 in THF solution and a picture of the related samples (inset) in natural light and under UV lamp at 365 nm are shown. The yellow THF solutions emit in the lime-yellow and the red region, respectively, with a negligible solvatochromic effect depending on the solvent polarity. PLQYs (see Table 1) have been measured in THF solution by relative methods using as standard quinine sulfate for A1 [41] and zinc phthalocyanine for A2 [42]. The solutions are stable and retain their optical characteristics up to three months under natural light at room temperature.

**Figure 2:**
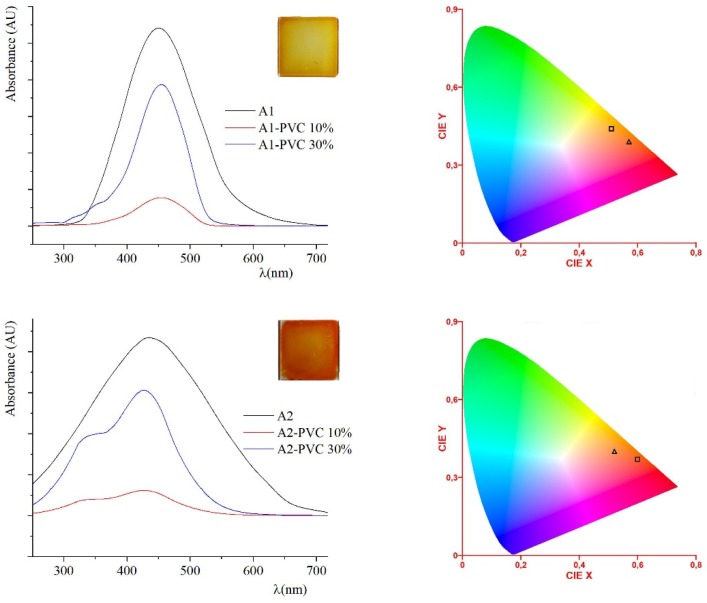
On the left: absorption curves of A1 (black curves), A1-PVC 10% (red curve) and A1-PVC 40% films (blue curve) above; absorption curves of A2 (black curves), A2-PVC 10% (red curve) and A2-PVC 30% films (blue curve) below. In the insets: 30% A1-PVC (above) and 30% A2-PVC (below) thin films. On the right: CIE diagram of A1 (triangle) and both its PVC blends (square) above; CIE diagram of A2 (triangle) and both its PVC blends (square) below.

The spin-coated thin films (obtained as described in the Materials and Methods section) of crystalline A1 and A2 have a red and yellow-orange color, respectively (see CIE in Table 1). Images of the spin-coated crystalline films of neat A1 and A2 under polarized microscope are reported in the Supplementary Materials, in Figure S3. The absorption spectra are reported in Figure 2, compared with the spectra of PVC blends at different dopant percentage. PVC as an economical and versatile thermoplastic colorable polymer is an excellent candidate to test the two compounds as dyes. It is the world’s third largest thermoplastic material widely used in construction, packaging, devices, and the textile industry. The demand for stable dyes for this white material is still high. The doped PVC homogeneous amorphous blends are an example of stable dyed blends with pigments at 10% and 30% by weight. In both cases, the dyes are soluble up to 30%. The diethylamino terminal groups make compound A1 more soluble than the dinitro derivative; hence A1 has a higher solubility limit (40%) in PVC. The PVC films have CIE coordinates in the orange-red region (see Figure 2), more red-shifted for A2-PVC, the same behavior recorded for THF solutions. The PVC films of blended A1 and A2 kept over three months in the air under natural light at room temperature perfectly retain transparency and optical characteristics. No swelling nor release of the same films were detected on samples kept in distilled water for 30 days at room temperature.

**Figure 3:**
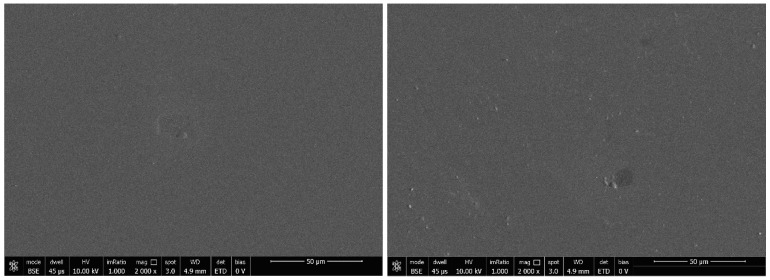
SEM image (2000x magnitude) of 30% A1-PVC film deposed onto quartz slide and metalized by Au-Pd sputtering (about 5 nm) (on the left). The same sample scanned after three months, on the right.

In Figure 3, an SEM image of 30% A1-PVC film deposed onto quartz slide is reported. The SEM analysis confirms that there is no visible structuring in the spin-coated film, also after three months under natural light at room temperature. All PVC blended samples of A1 and A2 exhibit similar morphological characteristics.

**Figure 4:**
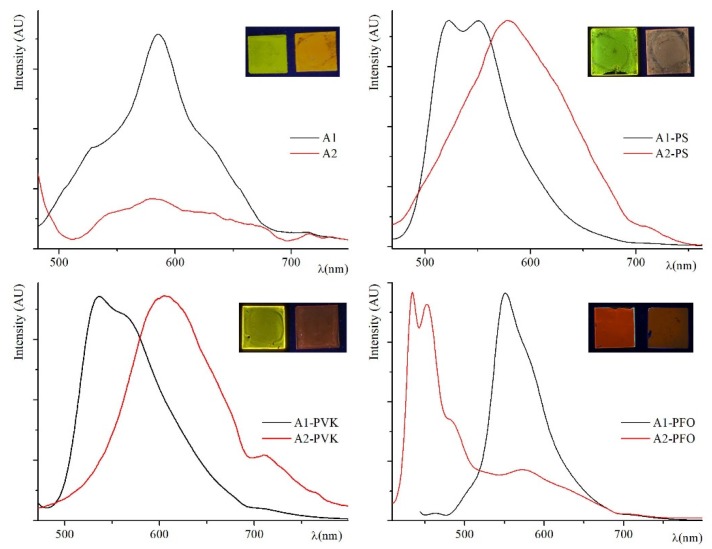
Emission spectra of the neat A1 (black curves) and A2 (red curves) samples (**a**), of their PS blends (**b**), PVK blends (**c**), and PFO blends (**d**). In the inset, the same spin-coated samples used for the measurements (A1 on the left and A2 on the right).

As for PL performance of neat A1 and A2, the crystalline dyes emit in the orange-red region with very similar maxima, see Table 2. The Stokes Shifts are about 140 nm. Appreciable PLQYs (see Table 2) measured on the crystalline samples spin-coated on quartz slides are a good result for azobenzene dyes. The PL response for A1 is higher than the nitro derivative A2 as a result of the different conjugation patterns, as discussed in the DFT analysis section. In Figure 4a, the emission spectra of the crystalline dyes are reported, and their PL behavior will be discussed in the next section.

### 2.2. PL Properties of PS, PVK, and PFO Blends

Polymeric blends are a well-known approach leading to an increase in the emission ability. Blended active layers have been demonstrated to be advantageous to fabricate optoelectronic devices such as efficient pure-color LEDs [43,44,45], guaranteeing easiness of fabrication, high processability, and low-cost. This situation resembles a diluted solid solution of the emitters into amorphous domains preventing aggregation caused quenching (ACQ) effect [46,47]. Different doped films were spin-coated by dissolving the dyes in a non-emissive and non-conductive (PS) or an emissive and conductive (PVK and mostly PFO) amorphous polymeric matrix. These polymers are typically employed in the construction of emissive layers. In natural light, the doped layers are homogeneous and transparent films except for A2-PFO. They display various shades of color, from yellow to red. Different dye percentages were tested in order to get better PL performance. As expected on the base of our previous study [48,49,50,51], the best emission for both samples was recorded on the more diluted blends (10 wt%), where the ACQ effect is attenuated. 

The films turned out to be stable in air for three months at room temperature under natural light, showing identical optical properties both in absorption and emission. In fluorescence, broad emission bands peaked between orange and orange-red are recorded, with Stokes Shift values ranging from 83 to 183 nm. The maximum of the absorption and emission bands of the blends are reported in Table 2.

In Figure 4, the emission spectra of the blend samples are compared with the crystalline ones. The emission spectra of pure PVK and PFO are reported, for comparison, in Figure S4 of Supplementary Materials section.

A2-PVK blend is the most red-shifted emitter with CIE (0.55; 0.44) (see Table 2). Quantitatively, all A1 blends show higher PLQYs respect to A2 blends but in the case of PFO samples, clearly highlighting the difference in the electronic pattern of the two chromophores. In both cases, PLQYs of the PS blends are lower respect to the analogous PVK blends. For an azobenzene material, the PL performance of A1-PVK is a remarkable achievement. As PVK is poorly fluorescent, 40% PLQY is due mostly to the chromophore itself as the ACQ effect is suppressed. The PL performance of the A1-PFO blend is particularly interesting. Polyfluorene and its derivatives (PFs) have recently emerged as the most promising polymeric matrixes due to their PL and electroluminescence efficiencies, good thermal and chemical stability [52]. Poly (9-octylfluorene) itself emits blue light with a large bandgap. The typical approach to realize tuned emission from polyfluorene derivatives is based on the copolymerization of low-bandgap π-conjugated moieties with PFO monomers. Unfortunately, polyfluorides often show both excimer and aggregate formation during thermal annealing. The formation of excimers involves the generation of dimerized units of the polymer that emit light at energies lower than those of the polymer itself. This effect hinders the use of polyfluorenes for most applications [53].

In our case, the simple approach to dissolve a red/orange dopant in PFO produced an efficient (57% PLQY) bright orange/red (see CIE coordinates in Table 2) luminescent blend. The outstanding PL performance of A1-PFO is due to the excellent match between the chromophore and the polymeric matrix, as rationalized by DFT calculations in the Supplementary Materials part. On the contrary, the attempt to homogeneously dissolve A2 in PFO failed. Only in that case, the film was not perfectly homogeneous and transparent, and the emission of the chromophore in the red region is negligible compared to the much higher blue emission of PFO itself (see Figure 4d and Figure S4 in Supplementary Materials).

### 2.3. DFT Analysis 

**Figure 5:**
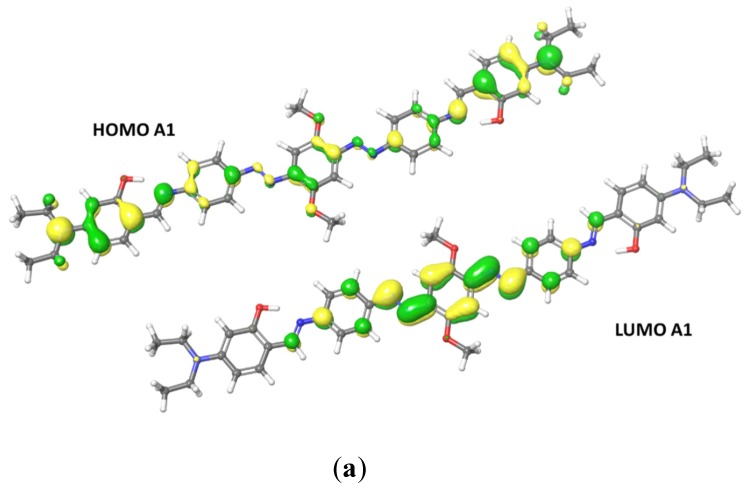

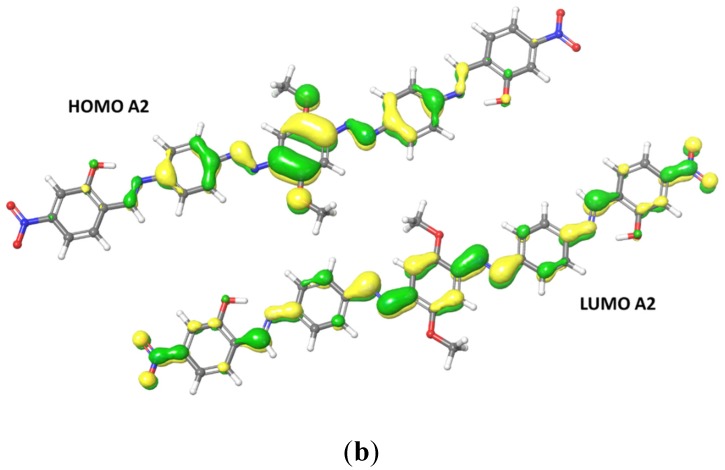
Frontiers orbitals HOMO and LUMO calculated for A1 (**a**) and A2 (**b**).

TDDFT approach at DFT level, using adiabatic local density approximation and ethanol as the simulated solvent, was used to run excitation energies calculations. Table 3 shows the most relevant optoelectronic properties calculated for the compounds A1 and A2. For A1, HOMO delocalization covers the entire conjugated backbone, extending to the terminal groups. LUMO is delocalized over the central rings and diazo groups. The main transition for both compounds is HOMO → LUMO, at 425 nm for A1 and 419 nm for A2. For A2, HOMO is mainly delocalized over the central ring and the oxygen atoms (Figure 5). A2 LUMO shows the electron-withdrawing effect of the nitro groups, with a higher delocalization of the orbitals over the terminal groups compared to the diethylamino derivative A1. The HOMO-LUMO gap is also higher for A2 than for A1 (Table 3). Because of the electron-withdrawing effect of the nitro groups, A2 shows a higher oxidation potential compared to A1, whereas the hole and electron reorganization energies (HRE and ERE) are typical for these class of compounds. A2 shows a decrease in the ERE due to the presence of the nitro groups. Reorganization energy (RE) is one of the parameters involved in the hopping rate, and HRE and ERE are strongly correlated to cation and anion geometries. A compound with a small RE usually shows high carrier mobility, and the energies are proportional to the deformation of the geometry during the process of charge transfer. Both derivatives show an ERE higher than their HRE, which means that there is less deformation upon electron injection compared to hole injection. Furthermore, the nitro derivative shows a smaller electron extraction potential, meaning that electron injection into A2 is easier than into A1. The electron-withdrawing effect of the nitro groups affects the electron-transporting features of the system.

The significant PL performance of A1 blended in PFO with 57% PLQY must be underlined. This result appears striking, keeping in mind that PFO itself is a blue emitter and A1-PFO blend emit in the orange-red region. Moreover, this solid-state emission is due to an azobenzene dye. For this reason, we analyzed the behavior of A1 in the PFO blend, the entire discussion reported in the Supplementary Material section. The formation of stacked dimers of the azo dye and PFO resulted in modifying the electro-optical properties significantly with the lowering of HOMO and LUMO values for both systems. The new HOMO energy values are even closer to the HOMO values of the PFO (Figure S1). Overall, stacking the dyes with PFO provides a shortcut for electronic transitions and is responsible for some degree of PFO distortion. Therefore, it must be underlined the ability of the azo dyes to modulate the optoelectronic characteristics of the PFO through two effects: the steric hindrance operated by the octyl chains, and the stacking between the dyes and the polymer. Especially in the case of A1-PFO, the calculations show a significant reduction of the electronic hopping energy barrier and justify the high quantum yield observed. Analysis of the oxidation potentials of the substituted dyes revealed that the oxidation potential of A1 dyes was about 460 mV easier to oxidize (more negative potentials) than the A2. Analysis of the reduction potentials revealed that A2 was about 400 mV easier to reduce (more positive potentials) than A1 dye. The diminished ability to reduce A1 dye is attributed to the presence of electron-withdrawing groups. Compared with other azo dyes like the 4-methoxylazobenzene [54] with a E_red_ of −1.44 V, or diarylaminoazobenzenes with a reduction potential between −1.36 V and −1.50V [55], both A1 and A2 have a lower reduction tendency and higher stability (see Tables S1 and S2 in Supplementary Material).

## 3. Materials and Methods 

Commercially available starting products were supplied by Sigma Aldrich (Sigma-Aldrich Corporation, St. Louis, MO, USA). 2-hydroxy-4-nitrobenzaldehyde was obtained as described in [56]. 4,4′-((1,1′)-(2,5-dimethoxy-1,4-phenylene)bis(diazene-2,1-diyl))dianiline (AB-NH_2_) was obtained as described in [13]. ^1^H NMR spectra were recorded in DMSO-d_6_, with a Bruker Advance II 400 MHz apparatus (Bruker Corporation, Billerica, MA, USA). Mass spectrometry measurements were performed using a Q-TOF premier instrument (Waters, Milford, MA, USA) equipped by an electrospray ion source and a hybrid quadrupole-time of flight analyzer. 

Zeiss Axioscope polarizing microscope (Carl Zeiss, Oberkochen, Germany) equipped with an FP90 Mettler hot stage (Mettler-Toledo, LLC-Columbus, OH, USA). DSC/TGA Perkin Elmer TGA 4000 (PerkinElmer, Inc., Waltham, MA, USA), scanning rate 10 °C/min, provided phase transition temperatures and enthalpies. The decomposition temperatures (the temperature at 5 wt.% weight loss, T_d_) were measured under nitrogen flow. UV-Visible and fluorescence spectra were recorded by JASCO F-530 and FP-750 spectrometers (JASCO Inc., Mary’s Court, Easton, MD, USA). Thin films of the neat samples and the polymeric blends were obtained using an SCS P6700 spin coater (Specialty Coating Systems Inc., Indianapolis, IN 46278, USA) operating at 600 rpm for 1 min (first step) and at 1200 rpm for 1 min (second step). 10 wt% NMP solution of the chromophores in commercially available PS (molecular weight 18,700 Da), in PVK (molecular weight 1100 Da) or PFO (molecular weight ≥20,000 Da), were employed. 

Photoluminescence quantum yield (PLQY) measurements were conducted with a setup similar to that of de Mello et al. [57]. It considers not only the excitation laser and direct photoluminescence but also the scattering of the integrating sphere that is part of the setup. The setup consists of a 405 nm laser, whose emission does not overlap with the photoluminescence spectrum, an integrating sphere (Stellarnet Inc, Tampa, FL, USA) and a photo spectrometer (BLACK Comet Stellarnet Inc, Tampa, FL, USA). The emission was measured at five different points on the sample.

Field emission scanning electron microscopy (FESEM) images were obtained with a FEI Nova NanoSEM 450 emission SEM (Thermo Fisher Scientific Inc., Waltham, MA, USA) at an accelerating voltage of 10 kV (range of acceleration voltage: 50 V–30 kV) equipped with an Everhart Thornley detector (ETD) and a Through Lens Detector (TLD). Samples were mounted on Al specimen mounts and coated with a thin layer of Au-Pd in order to eliminate any undesirable charge effects during the SEM observations.

### 3.1. Synthesis of A1 and A2

The same general procedure was employed for the synthesis of A1 and A2. The synthesis of A1 is described as an example. To 3.76 g (0.01 mmol) of AB-NH_2_ [39] dissolved at 70 °C in 30 mL of dry THF 3.87 g (0.02 mmol) of 4-(diethylamino)-2-hydroxybenzaldehyde was added under stirring. After 1 h, the crude product precipitated at room temperature. The compound was crystallized by dichloromethane/hexane and furtherly purified by washing with hot acetone. T_m_ = 280 °C; T_d_ = 330 °C. ^1^H NMR (400 MHz, DMSO *d*_6_, 25 °C, ppm): 1.12 (t, 12H), 3.46 (m, 8H), 4.00 (s, 6H), 6.21 (d, 4H), 6.44 (d, 2H), 7.46 (m, 8H), 7.65 (m, 2H), 8.89 (s, 2H), 10.65 (s, 2H). Elemental analysis calculated (%) for C_42_H_46_N_8_O_4_: C, 69.40; H, 6.38; N, 15.42; found: C, 69.60; H, 6.88; N, 15.88. MALDI-TOF of A1 m/z: 727.39 (M + H).

Chromophore A2 was obtained using 1,1,2,2-tetrachloroethane as the solvent and furtherly purified by washing with hot acetone and then hot dioxane: T_m_ = 327 °C; T_d_ = 330 °C. ^1^H NMR (400 MHz, DMSO-d_6_, 25 °C, ppm): 4.14 (s, 6H), 6.65 (m, 4H), 7.20–8.80 (m, 12 H), 9.20 (s, 2H), 12.55 (s, 2H). Elemental analysis calculated (%) for C_34_H_26_N_8_O_8_: C, 60.53; H, 3.88; N, 16.61; found: C, 60.50; H, 3.09; N, 16.80. MALDI-TOF of A2 m/z: 675.16 (M + H).

### 3.2. Theoretical Calculations

Quantum-mechanical calculations were performed with the Jaguar package, Schrödinger Release 2017-4 [58], on the theoretical level DFT/B3LYP, and the molecular geometry was optimized with functional B97-D3 [59]. Charges were determined using the NBO approach. Dunning’s correlation-consistent triple-ζ basis set cc-pVTZ (-f), which includes a double set of polarization functions, was used for single-point calculations on optimized geometries. TD-DFT and Tamm-Dancoff [60] approximations were used to perform calculations at neutral compound geometry to extract absorption values from vertical excitation energies. The solvent was simulated using Poisson Boltzmann Solver (PBF) [61]. The stacked dimers shown in Figure S2 were constructed with the Jaguar package from the minimized structures. Each dimer was initially minimized by simulated annealing using AMBER15FB [62] forcefield and then optimized at the B3LYP level, maintaining the dimer starting structure. 

Computed redox data was used to calculate “scaled” HOMO and LUMO energies, through the following equations:

Absolute Electrode Potential = Electrode Potential + NHE Energy(1)

Orbital Energy = Redox Potential + Absolute Electrode Potential(2)

where “NHE Energy” represents the energy of the NHE electrode in water (−4.28 V) and “Electrode Potential” represents the potential of the chosen electrode relative to NHE.

## 4. Conclusions

The spectroscopic behavior of two novel azobenzene chromophores with a symmetrical skeleton was systematically examined. Their coloring ability was tested in solution as neat crystalline samples and dissolved in a polymeric amorphous matrix. The doped PVC blends are an example of stable dyed polymers. In both cases, the dyes A1 and A2 are soluble up to 30 wt% in PVC matrix with CIE coordinates in the orange to red region. The films of both neat and blended dyes kept over three months under natural light at room temperature in the air perfectly retain structural and optical characteristics. No swelling nor release was detected in distilled water for 30 days.

On the other hand, the effort to obtain good steady PL response from azobenzene scaffolds is justified by the poor scientific documentation about this versatile and tunable functional group. A systematic study of the optical performance of the two differently substituted azobenzene dyes was performed, and DFT computational study gave information on HOMO-LUMO localization. The azo-based chromophores are emissive in solution and the solid-state, claiming a role as dye-dopants for emissive layers. Low-doped blends were obtained in PS, PVK, and PFO. PLQYs measured on the emissive blends are above 10% in all cases. In particular, PLQY of the orange-red A1-PFO blend (57%) represents a remarkable result for azobenzene based material, able to modulate the optoelectronic characteristics of the blue emissive PFO. The simple synthetic procedure, the affordability, solubility and processability of the dyes and the doped blends make the azo-dyes rare examples of azobenzene based materials potentially employable both as dyes and as fluorophores.

## Figures and Tables

**Table 1:** Optical data for A1 and A2 in solution and for polyvinyl chloride (PVC) blends.

Sample	λ_abs_ (nm) ^a^	ε (10^5^ cm^−1^M^−1^) ^b^	λ_em_ (nm) ^c^	PLQY% ^d^	λ_abs_ (nm) ^e^	CIE ^f^
**A1**	448	6.29	511 (542)	3.0 ± 0.1	444	0.57; 0.39
**A2**	(348) 453	9.57	619	1.2 ± 0.1	435	0.52; 0.40
**A1-PVC(10%)**	-	-	-	-	455	0.51; 0.44
**A2-PVC(10%)**	-	-	-	-	455	0.60; 0.37
**A1-PVC(30%)**	-	-	-	-	426	0.51; 0.44
**A2-PVC(30%)**	-	-	-	-	425	0.60; 0.37

^a^ Wavelength of UV-Visible absorbance maxima in THF solution. ^b^ Molar absorption coefficient. ^c^ Wavelength of emission maxima in THF solution. ^d^ PLQYs in THF solution. ^e^ Wavelength of UV-Visible absorbance maxima measured on the spin-coated film. ^f^ Absorption CIE coordinates on the spin-coated film.

**Table 2:** Optical data for A1 and A2 as neat solid samples and in 10 wt%. poly (styrene) (PS), poly (vinylcarbazole) (PVK), and poly (9,9-dioctylfluorene) (PFO) film blends.

Blend	λ_abs_ (nm) ^a^	λ_em_ (nm) ^b^	PLQY% ^c^	CIE ^d^
**A1**	444	585	3.4 ± 0.1	0.48; 0.50
**A2**	435	578	0.7 ± 0.1	0.50; 0.49
**A1-PS**	439	523–551	22.0 ± 0.1	0.35; 0.60
**A2-PS**	415	578	11.0 ± 0.1	0.47; 0.49
**A1-PVK**	440	536	40.0 ± 0.4	0.41; 0.56
**A2-PVK**	444	605	10.0 ± 0.1	0.55; 0.44
**A1-PFO**	390	551	57.0 ± 0.5	0.43; 0.50
**A2-PFO**	390	434, 452, (571)	63.0 ± 0.7	0.23; 0.19
**PS ^e^**	290	-	-	-
**PVK ^e^**	295, 340	388	7.6 ± 0.5	0.20; 0.16
**PFO ^e^**	365	459	68.0 ± 0.4	0.20; 0.24

^a^ Wavelength of UV-Visible absorbance maxima; ^b^ Wavelength of emission maxima; ^c^ Photoluminescent quantum yield; ^d^ Emission CIE coordinates; ^e^ Optical data for films of PS, PVK, and PFO, obtained in the same conditions as the blends.

**Table 3:** Electro-optical properties calculated on A1 and A2 in vacuum.

Properties	A1	A2
**Oxidation Potential (eV)**	0.53	1.01
**Reduction Potential (eV)**	−1.08	−0.76
**Hole Reorganization Energy (eV)**	0.31	0.33
**Electron Reorganization Energy (eV)**	0.40	0.35
**λ_max_ (nm)**	425	419
**E_max_ (nm)**	540	538
**Scaled HOMO (eV)**	−4.98	−5.45
**Scaled LUMO (eV)**	−2.26	−2.69
**HOMO-LUMO (eV)**	2.72	2.76

## Supplementary Materials

Table S1. Electro-optical properties calculated on A1 and A2 in vacuum and associated with PFO. Table S2. Reduction potential of similar azobenzenes [2,3]. Figure S1. Energies of orbital levels of PFO, A1 and A2. The arrows show possible electronic transitions. Figure S2. Frontiers orbitals HOMO and LUMO calculated for the dimers PFO-A1 (above) and PFO-A2 (below). Figure S3. Pictures of A1 and A2 crystalline films under polarized light. Figure S4. Emission spectra of PVK (orange line) and PFO (blue line) films, excited on the absorption maxima.
