# Supplementary material for: Spectroscopic Behaviour of Two Novel Azobenzene Fluorescent Dyes and Their Polymeric Blends
Source: Molecules. 2020 Mar 17;25(6):1368. doi: 10.3390/molecules25061368 (PMC7144390; doi:10.3390/molecules25061368)
Supplement: Supplementary file 1 [file molecules-25-01368-s001.pdf]

Spectroscopic Behaviour of Two Novel Azobenzene Fluorescent Dyes and Their Polymeric Blends

Rosita Diana[1], Ugo Caruso[2*], Stefano Piotto[3], Simona Concilio[4], Rafi Shikler[5] and Barbara Panunzi[1]

[1] Department of Agriculture, University of Napoli Federico II, Portici NA, Italy
[2] Department of Chemical Sciences, University of Napoli Federico II, Napoli, Italy
[3] Department of Pharmacy, University of Salerno, Fisciano SA, Italy
[4] Department of Industrial Engineering, University of Salerno, Fisciano SA, Italy
[5] Department of Electrical and Computer Engineering, Ben-Gurion University of the Negev, POB 653 Beer-Sheva 84105 Israel
* Correspondence: ugo.caruso@unina.it; Tel.: +39 081 674366

Supplementary Materials

DFT analysis of A1 and A2 PFO blends

As for the PFO blends, absorption and emission maxima are summarized in Table S1. The absorption of the azo molecules, around 430 nm is masked by the polymer signal at 390 nm. In emission, the two peaks at 434 and 452 are due to PFO and the peaks at 551 and 571 are related to the dyes. The PFO itself is an optically active material. In the presence of the azobenzene dye it distorts in order to interact with the molecules and the energy level and spatial proximity allow a hopping between the two systems as shown in Figure S1.

When it is possible to achieve the same type of hopping already observed in similar systems [1] PLQYs are particularly high. In Figure S1, it can be observed how the excitation of the PFO allows the hopping on the LUMO of A1 and A2, with consequent emission 3a and 3b. PFO itself has a wide range of wavelengths in absorption and emission. In fact, the completely minimized and planar PFO corresponds to an absorption with wavelengths of about 540 nm but the distortion caused by the octyl chains reduces the absorption to less than 400 nm. A1 and A2 added to the polymer matrix in mass percentages of 10% act as an additional thinner and increase their own quantum yield. An A1 or A2 molecule approximately has the length of four fluorene units. Therefore, a mixture of azo dyes with PFO in a ratio of 1: 9 results in the availability of about ten fluorene units for A1 or A2. The ratio is almost optimal to ensure stoichiometric interaction between the two systems provided that the systems have the right miscibility. Using the module Blends of the suite BIOVA Materials Studio 2017 (17.1.0.48) Copyright© 2016 Dassault Systémes, we evaluated the mutual miscibility of A1 and A2 in PFO, and A1 results having a mixing energy of circa 0.8 kcal/mol lower than A2. A difference of less than 1 kcal/mol can be responsible of aggregates of A2 not perfectly dissolved as observed in the main text of the article.

Table S1. Electro-optical properties calculated on A1 and A2 in vacuum and associated with PFO.

Properties	A1	A2	A1-PFO	A2-PFO
**Oxidation Potential (eV)**	0.53	1.01	0.56	1.02
**Reduction Potential (eV)**	-1.08	-0.76	-1.12	-0.75
**Hole Reorganization Energy (eV)**	0.31	0.33	0.35	0.37
**Electron Reorganization Energy (eV)**	0.40	0.35	0.45	0.50
**λ_{max} (nm)**	425	419	420	425
**E_{max} (nm)**	540	538	530	539
**Scaled HOMO (eV)**	-4.98	-5.45	-5.02	-5.40
**Scaled LUMO (eV)**	-2.26	-2.69	-2.26	-2.68
**HOMO-LUMO (eV)**	2.72	2.76	2.76	2.72

Table S2. Reduction potential of similar azobenzenes [2,3]

name	Experimental Reduction potential (V)	Calculated Reduction potential (V)
**4-methoxyl (E)-1,2-diphenyldiazene**	-1.44	-1.40
**(E)-N,N-diphenyl-4-(phenyldiazenyl)aniline**	-1.50	-1.42
**(E)-4-methyl-N-(4-(phenyldiazenyl)phenyl)-N-(p-tolyl)aniline**	-1.42	-1.32
**(E)-4-methoxy-N-(4-methoxyphenyl)-N-(4-(phenyldiazenyl)phenyl)aniline**	-1.44	-1.30

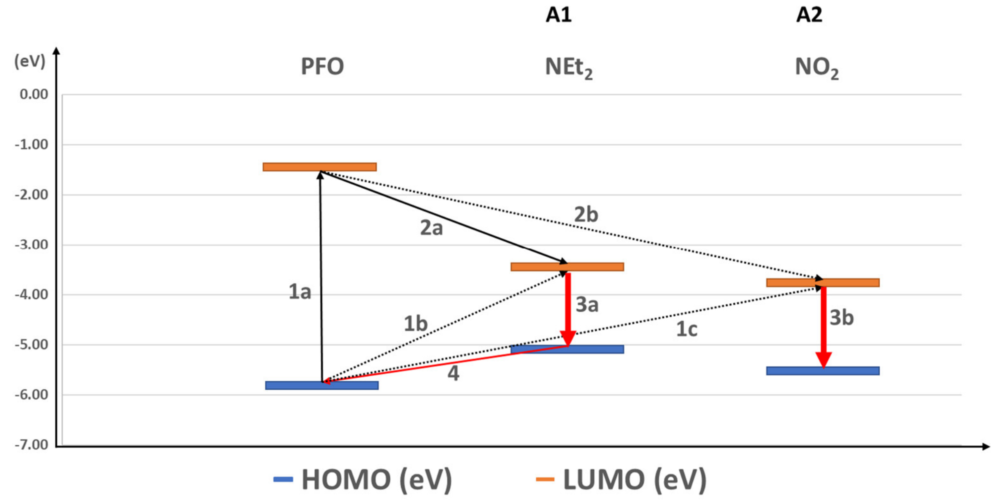

Figure S1. Energies of orbital levels of PFO, A1 and A2. The arrows show possible electronic transitions.

The effect of stacking on fluorescent properties of both PFO blends was evaluated by time-dependent density functional theory (TDDFT). The excited-state properties of the two molecules both isolated and stacked with PFO (dimers) were calculated and the most relevant electro-optical properties summarized in Table S1.

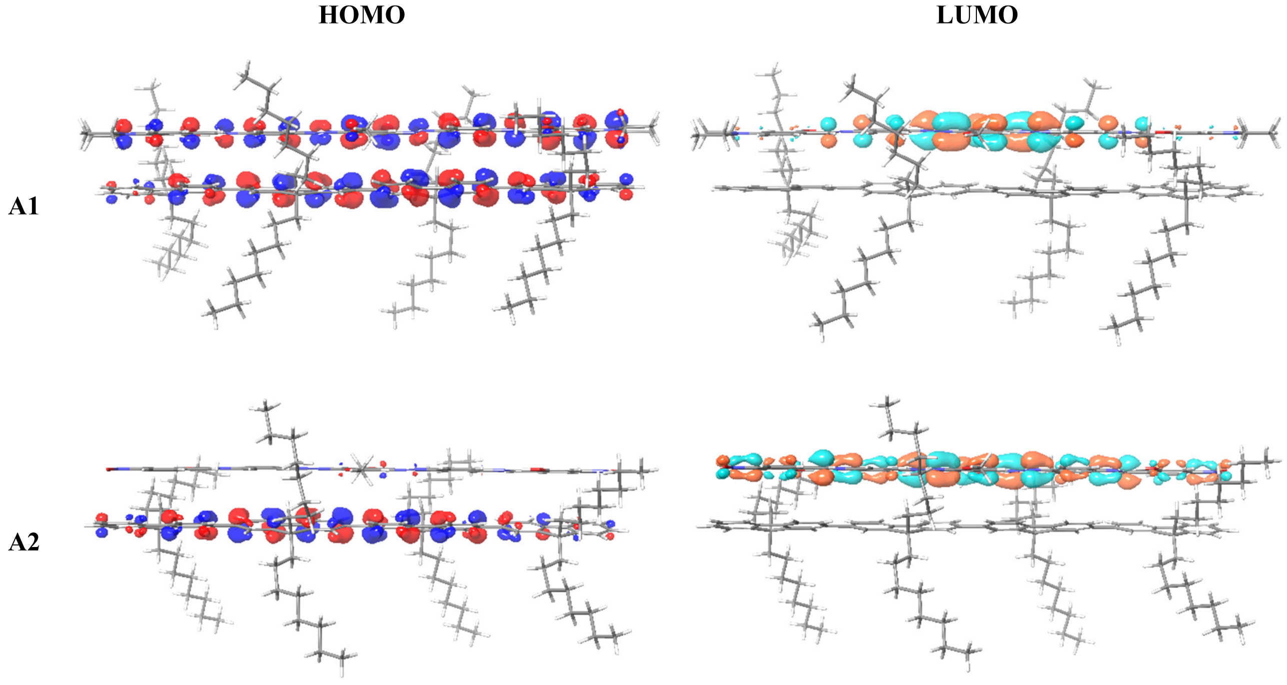

Figure S2. Frontiers orbitals HOMO and LUMO calculated for the dimers PFO-A1 (above) and PFO-A2 (below).

Because both PFO and azo dyes are conjugated π-systems, different stacking geometries that alter the extent of base overlap are predicted to change their electronic properties.

LUMO is located on the azo-molecule and HOMO mainly on the PFO. It is interesting that in the case of the dimer PFO-A1, HOMO is extended on both molecules. The phenomenon is well known and typical in aminopurines [4]. This spatial proximity reduces the difficulty of electronic hopping and increases the PL quantum yield. As summarized in Table S1 and shown in Figure S2, the formation of stacked dimers of the azo dyes and PFO modifies significantly the electro-optical properties. Noteworthy is the lowering of HOMO and LUMO values for both systems. The new HOMO energy values are closer to the PFO HOMO values. A shortcut for electronic transitions due to stacking the dyes with PFO is responsible for some distortion in the matrix. A significant reduction of the electronic hopping energy barrier is recorded in the case of PFO-A1 which shows the high (57%) quantum yield.

Crystalline films of A1 and A2

Thin films of crystalline A1 and A2 have been obtained by spin coating, and they have a red and yellow-orange color, respectively. To asses the crystallinity of the films, they were observed under a polarized light microscope. Figure S3 shows the crystalline layer of the two films of A1 and A2 under polarized light.

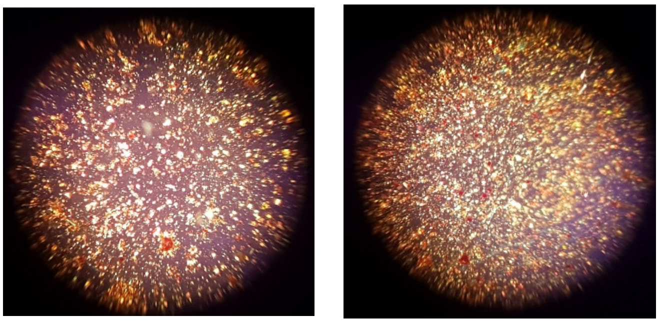

A1 **A2**

Figure S3. Pictures of A1 and A2 crystalline films under polarized light.

PL properties of PS, PVK, and PFO films

Doped films were spin-coated by dissolving the dyes A1 and A2 in PS, PVK and PFO amorphous polymeric matrices, and the optical properties of the blends were studied. The best emission for both samples was recorded on the more diluted blends (10 wt%). For comparison, in Figure S4 we report the emission spectra of the pure polymers PVK and PFO, in thin films. PS is a non-emissive polymer.

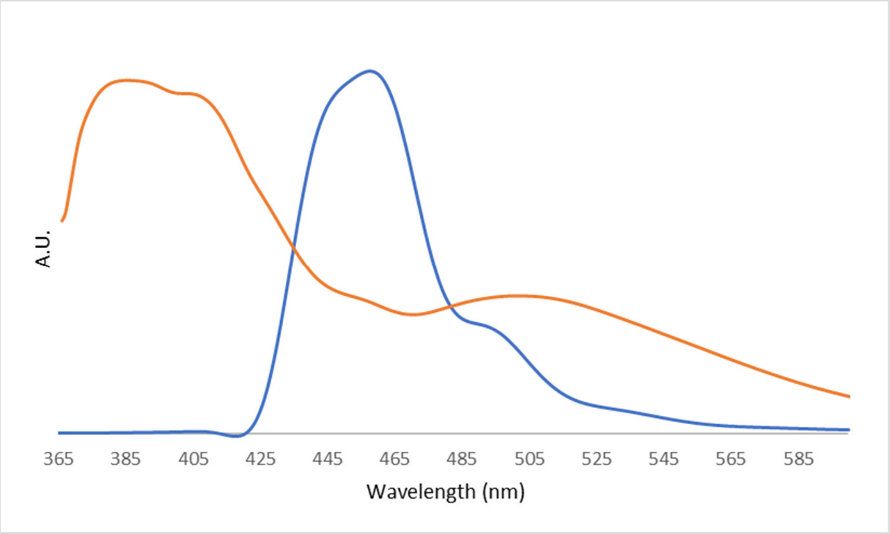

Figure S4. Emission spectra of PVK (orange line) and PFO (blue line) films, excited on the absorption maxima.

1. Ahlström, L.-H.; Sparr Eskilsson, C.; Björklund, E. Determination of banned azo dyes in consumer goods. *TrAC Trends in Analytical Chemistry* **2005**, *24*, 49-56, doi:10.1016/j.trac.2004.09.004.
2. Chiu, K.Y.; Tran, T.T.H.; Wu, C.-G.; Chang, S.-H.; Yang, T.-F.; Su, Y.O. Electrochemical studies on triarylamines featuring an azobenzene substituent and new application for small-molecule organic photovoltaics. *Journal of Electroanalytical Chemistry* **2017**, *787*, 118-124.
3. Chiu, K.Y.; Tu, Y.-J.; Lee, C.-J.; Yang, T.-F.; Lai, L.-L.; Chao, I.; Su, Y.O. Unusual spectral and electrochemical properties of azobenzene-substituted porphyrins. *Electrochimica acta* **2012**, *62*, 51-62.
4. Pedersen, T.G.; Johansen, P.M.; Pedersen, H.C. Characterization of azobenzene chromophores for reversible optical data storage: molecular quantum calculations. *Journal of Optics A: Pure and Applied Optics* **2000**, *2*, 272-278, doi:10.1088/1464-4258/2/4/305.
